# Achieving stomal continence with an ileal pouch and a percutaneous implant

**DOI:** 10.1007/s10856-021-06633-4

**Published:** 2022-01-04

**Authors:** Martin L. Johansson, Leif Hultén, Olof Jonsson, Heithem Ben Amara, Peter Thomsen, Bjørn Edwin

**Affiliations:** 1 Department of Biomaterials, Institute of Clinical Sciences, Sahlgrenska Academy, University of Gothenburg, Gothenburg, Sweden; 2 Department of Surgery, Sahlgrenska University Hospital, Gothenburg, Sweden; 3 Department of Urology, Institute of Clinical Sciences, Sahlgrenska Academy, University of Gothenburg, Gothenburg, Sweden; 4 The Intervention Centre, Rikshospitalet, Oslo University Hospital, Oslo, Norway; 5 Department of Hepato-Pancreato-Biliary Surgery, Rikshospitalet, Oslo University Hospital, Oslo, Norway; 6 Institute of Clinical Medicine, University of Oslo, Oslo, Norway

## Abstract

In this study, a soft-tissue-anchored, percutaneous port used as a mechanical continence-preserving valve in reservoir ileo- and urostomies was functionally and morphologically evaluated in eight dogs. During follow-up, the skin failed to attach to the implant, but the intestine inside the stoma port appeared to be attached to the mesh. After reaching adequate reservoir volume, the urostomies were rendered continent by attaching a lid to the implant. The experiments were ended at different time intervals due to implant-related adverse events. In only one case did the histological evaluation reveal integration at both the implant-intestine and implant-skin interfaces, with a low degree of inflammation and the absence of bacterial colonisation. In the remaining cases, integration was not obtained and instead mucosal downgrowth and biofilm formation were observed. The skin-implant junction was characterised by the absence of direct contact between the epidermis and the implant. Varying degrees of epidermal downgrowth, granulation tissue formation, inflammatory cell infiltration and bacterial growth and biofilm formation were prominent findings. In contrast, the subcutaneously located anchor part of the titanium port was well integrated and encapsulated by fibrous tissue. These results demonstrate the opportunity to achieve integration between a soft-tissue-anchored titanium port, skin and intestine. However, predictable long-term function could not be achieved in these animal models due to implant- and non-implant-related adverse events. Unless barriers at both the implant-skin and implant-intestine junctions are created, epidermal and mucosal downward migration and biofilm formation will jeopardise implant performance.

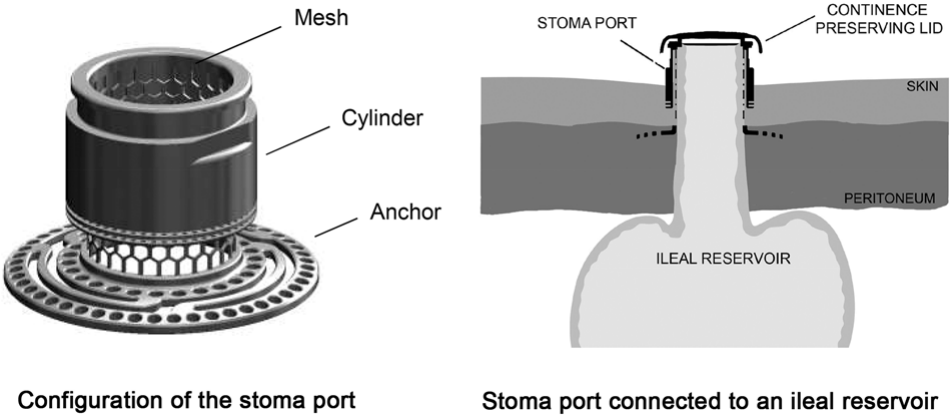

## Introduction

Inflammatory bowel disease, colorectal and gynaecological cancer, neurological bladder disorders and bladder cancer sometimes require excisional surgery with the creation of an abdominal stoma. Many attempts have been made over the years to overcome the problems of a conventional stoma by creating continent ileo- or urostomies, complex constructions often associated with considerable morbidity and failure rates [1–5]. The continent ileostomy, first described by Dr. Nils Kock in 1969, gained popularity in the 1970s and early 1980s, and the technique was also used for urinary diversion [6–8]. This method offers good continence in most cases, and patients enjoy a good quality of life. Nevertheless, the technique has lost its popularity, mainly due to the complexity of the procedure, high failure rate and frequent need for reoperation to restore continence, largely due to nipple valve malfunction. A number of methods to stabilise the nipple valve have been tried over the years, but the problem has not yet been satisfactorily solved, and the nipple valve is still the Achilles’ heel of both constructions [9–11].

Percutaneous, bone-anchored implants are being used increasingly in many applications for the treatment of disorders, the retention of prostheses and the administration of substances and energy through the skin [12, 13]. One particularly successful example is the bone-anchored hearing system (BAHS). Recent developments in surgical techniques and implant designs have improved the clinical outcome significantly compared with that of earlier generations of the system [14–17]. Despite these improvements leading to a survival rate of up to 98% in adult patients (mean follow-up time, 16 months), it is evident that adverse skin reactions remain a challenge in the use of these devices, with one of seven patients requiring treatment [18]. For bone-anchored limb prostheses, adverse skin reactions and osteomyelitis are even more pronounced [19–21]. In contrast to these clinically used bone-anchored devices, experience with soft-tissue-anchored, percutaneous implants is limited [22–24]. The use of a titanium implant to divert intestinal contents from the stoma is new, and until now, it has been unclear whether the intestinal wall will integrate with titanium or other synthetic, implantable materials.

In an attempt to replace the nipple valve used in the continent ileostomy, we developed a soft-tissue-anchored, percutaneous device to be used for creating a continence-preserving stoma port [25]. Anchorage of the exit conduit of the reservoir to the implant was obtained by integrating an inner titanium mesh structure inside the implant. A dog model was developed, and the implant was adapted to conduct longer studies with ileostomies and urostomies.

The aim of the present study in dogs was to explore the suitability of such a device to be merged with the outlet of an ileal reservoir and the surrounding abdominal wall and skin.

## Materials and methods

**Fig. 1:**
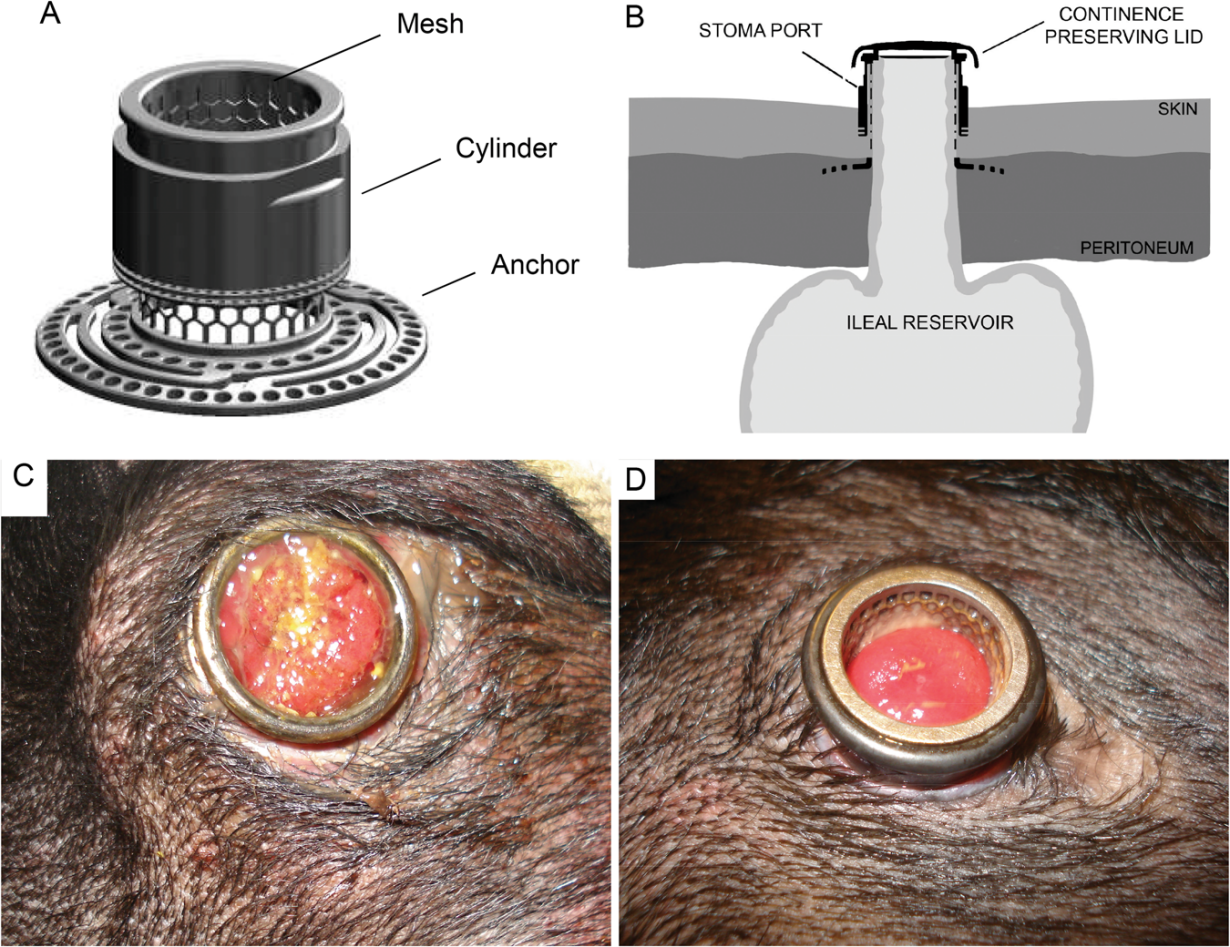
**A**, **B** Configuration of the stoma port and surgical models used. **A** The stoma port consisted of a skin-penetrating cylinder with an outer diameter of 20–22.5 mm and a ring-shaped anchor measuring 33–35.5 mm in diameter. The perforation of the anchor ring allowed connective tissue ingrowth. A cylindrical titanium mesh (inner diameter, 15–17.5 mm; hexagonal openings, 1.5 mm wide; wire width, 0.3–0.46 mm) was enclosed by the cylinder and attached to the anchor. **B** The stoma model with the titanium stoma port installed in the abdominal wall, the anchor flange located in the abdominal musculature, and the cylinder penetrating the skin. The ileal outlet from a valveless reservoir *ad modum* Kock was passed through the port. In a later stage, either the ileum or a ureter was anastomosed to the base of the reservoir. **C**, **D** Photographs of the stoma port implanted in dogs. **C** Ileostomy model after 6 weeks (EBM12) and **D** urostomy model after 26 weeks showing slight granulation around the implant and retraction of the efferent segment (EBM18)

A titanium stoma port was placed in the abdominal wall to act as a port for the outlet of an ileal reservoir. In the study, two different surgical models were employed: a continent ileostomy and a continent urostomy (Fig. 1A, B). Continence was sometimes preserved with a sealing lid on the implant.

### Implant designs

Cylindrical stoma ports with an internal mesh structure were fabricated from commercially pure grade 2 titanium (ISO 5832-2). The inner diameter of the stoma ports ranged between 15 and 17.5 mm and was sufficient to allow a dog’s small bowel to be conveniently pulled through. The cylinder was equipped with an inner titanium mesh to promote tissue ingrowth and secure anchorage of the reservoir outlet. For stabilisation in the abdominal wall, the mesh was mounted upon an anchor flange with penetrating circumferential tracks and multiple small holes to favour connective tissue ingrowth. The mesh was manufactured by laser cutting and electrochemically treated to remove any material debris. Prior to assembly using laser welding, the parts were blasted with aluminium oxide (grain size, 44–74 µm; KMC Ytbehandling AB, Järfälla, Sweden). Ports were ultrasonically cleaned and sterilised in an autoclave with a maximum temperature of 137 °C for 20 min. The test implants were manufactured by OstomyCure AS (Oslo, Norway). Two different surgical models were employed: an ileostomy reservoir and a urostomy reservoir. The configuration of the port is shown in Fig. 1A. A sealing lid placed on the top of the implant was occasionally used to achieve continence.

### Surface characterisation

The inside surface of the cylinder top and the mesh was subjected to chemical and morphological characterisation. The chemical composition of the surfaces was determined using Auger electron spectroscopy (AES) (PHI 660 Scanning Auger Microprobe) on an area of 55 × 45 μm with 5 scans in each area (Analyser resolution: 0.6 %, Primary beam energy: Ep = 10 keV, Spectrum width: 30–1100 eV, Beam current: *I* = 350 nA, Analysis time: 50 ms/eV). AES analysis combined with argon ion sputtering was used for analysing the thickness of the surface oxide (defined at which the oxygen signal has dropped to half its maximum value). The depth profiling was made using 3.5 keV Ar+-ions with a sputter rate of 25 nm/min and analysis parameters as for the chemical analysis. The sputter rate was calibrated using a Ta sample with known Ta_2_O_5_ oxide thickness. Scanning electron microscopy (SEM, Carl Zeiss Ultra 55) was used for imaging of the surfaces. All images were taken at magnifications ×100, ×1000 and ×5000 using 20 kV beam voltage. Analysis of surface contaminants was made using energy dispersive X-ray analysis (EDX). Surface topography was imaged, measured, and analysed using a non-contact optical profilometer using WYKO NT9100 optical profiler (Veeco Instruments Inc., Tucson, USA) and the Vision (version 4.10) software. The measurements were made over an arbitrary 155×116 μm area. Before calculating surface topography parameters raw data were processed with a tilt and if necessary cylindrical correction. A built-in median filter (3 × 3 pixel smoothing) for noise reducing was applied and if needed a mask was applied to block false data such as noise and spiky data points.

### Animal model

Eight female Labrador dogs (25–34 kg) were included in the study after 3 weeks of acclimatisation and training. The animals were housed according to local regulations. Postoperatively, each dog was fitted with a collar or protective jacket during the healing phase to prevent the dogs from disturbing the implant. The studies were conducted at the Department of Experimental Biomedicine, University of Gothenburg. The protocol of the study was approved by the regional Ethics Committee for Animal Research, Göteborg, Sweden (Dnr 325-2007).

### Anaesthesia

Animals were premedicated with an intramuscular injection of acepromazine 0.25 mg/kg, buprenorphine 0.02 mg/kg and propofol 4 mg/kg. Anaesthesia was induced with intravenous propofol and maintained by isoflurane inhalation. Respiration was secured by orotracheal intubation. Prophylactic antibiotics (amoxicillin/clavulanic acid, 8.75 mg/kg) were administered subcutaneously and continued for five days postoperatively. Analgesic treatment with buprenorphine and carprofen was given for a minimum of two days postoperatively. If the animals seemed to experience pain or discomfort after two days, they were treated with buprenorphine or carprofen orally. Experiments were terminated by an intravenous injection of pentobarbital followed by 40 mmol KCl.

### Surgical technique

A two-stage procedure was employed. In the initial surgical procedure, an isolated ileal reservoir was constructed, the stoma port was inserted in the abdominal wall, and the exit conduit of the reservoir was brought through the stoma port aperture. In the second surgical procedure, intestinal continuity was restored in four animals, creating a functional ileostomy, and in three animals, the left ureter was implanted in the reservoir, creating a functioning urostomy.

#### Construction of an ileal reservoir and installation of a port (*n* = 8)

The construction of an ileal reservoir with the installation of a titanium implant and the creation of a functioning reservoir ileo- or urostomy was performed as a two-stage procedure. A schematic view of the port installed in the abdominal wall and connected to the reservoir outlet is shown in Fig. 1B.

#### Stage I. Construction of an isolated ileal reservoir and installation of a titanium port

A midline incision was created in the abdomen. A 25-cm-long segment of the small intestine, taken 25–30 cm proximal to the ileocaecal valve, was isolated. The intestinal continuity was thereafter restored with side-to-side stapled anastomosis (ENDO GIA™ Universal 60 × 3.5, Autosuture, USA). Approximately 10 cm of the isolated segment was reserved for the future outlet. Two 8-cm limbs were sutured side to side and opened, and a pouch was created ad modum Kock (but without the intussuscepted nipple valve) [6]. The reservoir was then tested for leakage by filling the reservoir with 30–50 cc of air. From the midline incision, the anterior fascia of the rectus muscle was exposed. A short incision—creating a “buttonhole” opening and space for the implant’s flange—was made in the anterior fascia of the rectus muscle followed by an incision made using a trephine through the dermis/epidermis above the buttonhole. The flange of the implant was then inserted through the buttonhole, which was tightened using absorbable sutures (Vicryl 4–0); then, the titanium implant cylinder was passed through the skin. A channel was formed through the rectus muscle using diathermy and blunt dissection, and the ileal conduit was finally passed through the muscular wall and stoma port aperture. The reservoir was anchored to the peritoneum with two absorbable sutures. To prevent the port from retracting, a plastic ring was fastened on the external cylinder. After trimming the ileostomy to approximately 1–2 cm outside the implant, a catheter (18 or 20 Ch) was introduced into the reservoir and fixed in place with a suture. The abdomen was closed with interrupted sutures in the muscle fascia and mattress sutures in the skin. During the first two postoperative weeks, the reservoir was drained continuously by a catheter and flushed with saline a minimum of twice daily. After a median of 11 days (range, 4–21 days), the ileostomy stump was trimmed by diathermy approximately 5 mm above the implant cylinder. The twice-a-day flushing was continued, and graded distension of the reservoir was performed to increase its volume to approximately 60 ml.

#### Stage II. Establishment of a functioning reservoir ileostomy (*n* = 4) and urostomy (*n* = 3)

At varying intervals (median, 4.2 weeks; range, 3–10 weeks) after the first-stage procedure, the dogs were re-laparotomised. In four dogs, the terminal ileum was cut close to the ileocaecal valve, and the cut end was anastomosed to the ileal reservoir. In the remaining three dogs, the left ureter was identified and severed distally, and its cut end was anastomosed to the reservoir using a fish-mouth technique. A feeding tube was passed through the anastomosis, with its proximal end left outside the port. In addition, an indwelling balloon catheter was introduced through the port into the reservoir. In the urostomy group, the reservoir volume was thereafter further enlarged by gradually increasing the period of lid attachment to the implant (from 1 to 5 h). The implants were examined daily, and the condition of the operative field and local tissue was assessed. The degree of inflammation and occurrence of other adverse events were noted.

### Histological procedure and assessment

Implants with surrounding tissue were removed *en bloc* and immersed in 4% neutral-buffered formalin for one to two weeks. The specimens were dehydrated in increasing grades of ethanol and subsequently infiltrated and polymerised in LR White Resin (London Resin Company Ltd, Berkshire, UK). After embedding and polymerisation, the samples were cut using Exakt cutting-grinding equipment [26]. The final sections were approximately 50 µm thick. The implant with surrounding tissue was stained with Richardson’s stain before cover glass mounting. The morphological evaluation was performed by light microscopy using an Eclipse E 600 light microscope (Nikon, Kawasaki, Kanagawa, Japan) and connected computer software. The histological examination was performed on intact material-tissue preparations (ground sections). This enabled an analysis of the tissue in close contact with different portions of the port. Particular focus was directed towards the morphology of soft tissue around the internal mesh inside the cylinder and the morphology at the skin-cylinder junction. Tissue adaptation in these areas was determined in terms of inflammation, bacterial growth, epidermal downgrowth and tissue integration.

### Backscattered electron scanning electron microscopy

Selected resin embedded blocks were polished using 800–2000 grit SiC paper. A stock solution of iodine in potassium iodide (Lugol solution, I_2_/KI, Sigma-Aldrich) was diluted 1:1 in ethanol, and pipetted (~1000 μL) directly onto polished resin embedded blocks for ~15 min. The stain was washed off with deionized water. Backscattered electron scanning electron microscopy (BSE-SEM) was performed in a Quanta 200 environmental SEM (FEI Company, The Netherlands) operated at 20 kV accelerating voltage, 1 Torr water vapour pressure, and 10 mm working distance.

## Results

### Surface characterisation

**Fig. 2:**
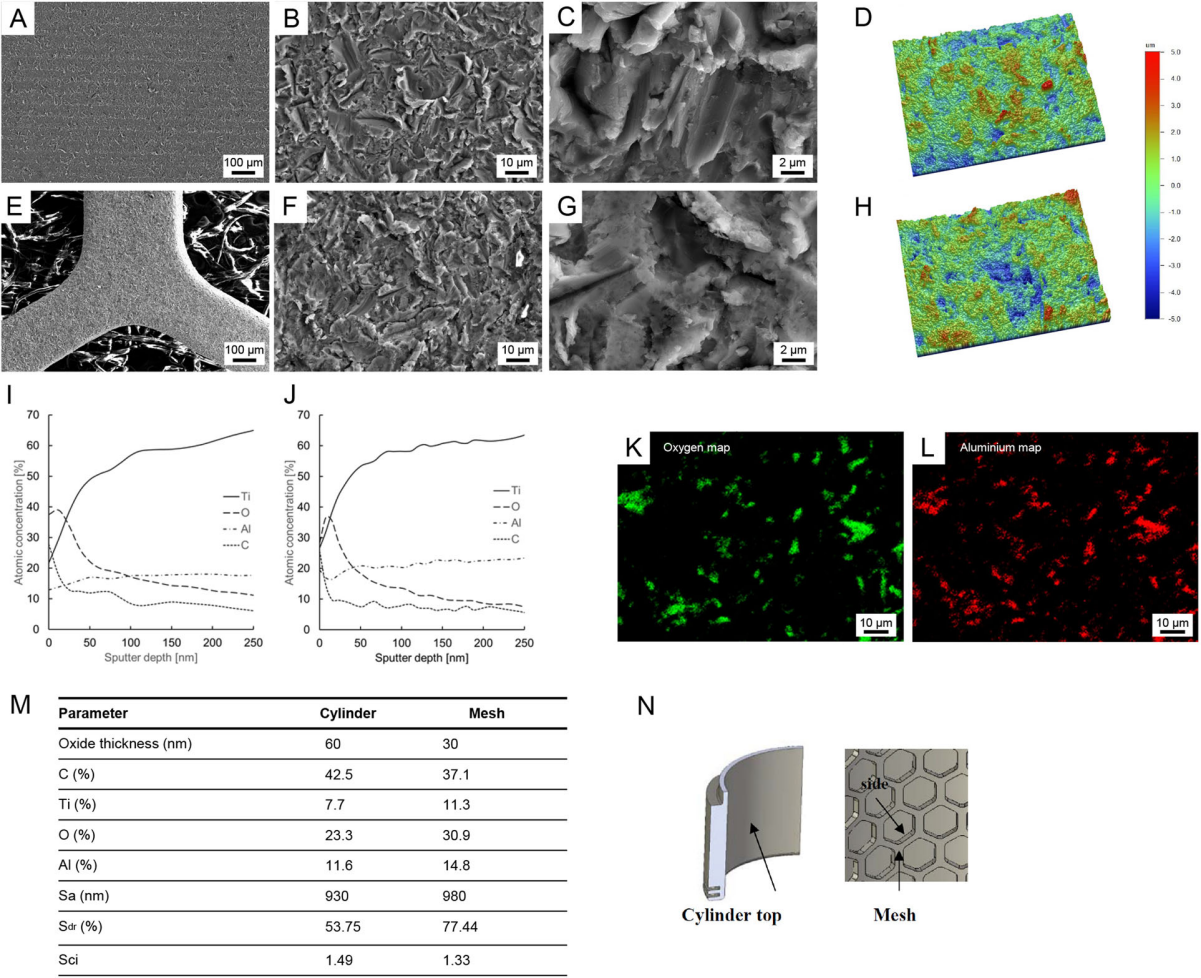
**A–N** Qualitative and quantitative topographical and chemical characterisation of the stoma port with detailed analysis of the cylinder and mesh. **A–D** Scanning electron micrographs and surface microtopography of the cylinder. **E**–**H** Scanning electron micrographs and surface microtopography of and the mesh. Auger electron spectroscopy (AES) depth profiling of the cylinder (**I**) and mesh (**J**). **K, L** Energy dispersive X-ray analysis (EDX) of the cylinder showing the oxygen and aluminium content in the surface. **M** Table showing the oxide thickness, chemical composition of the surface and the result from the surface topography measurements. Sa: arithmetic mean deviation of the surface; Sdr: developed surface area ratio; Sci: surface core fluid retention index. **N** Image showing the cylinder top (left) and mesh (right) where chemical and topographical data were obtained

Qualitative and quantitative topographical and chemical characterisation of the stoma port with detailed analysis of the cylinder and mesh is shown in Fig. 2. For the cylinder top, medium resolution SEM images revealed the typical banded feed marks in the form of ridges and grooves created during turning (Fig. 2A), whereas the mesh that was manufactured using laser cutting demonstrated a more uniform surface (Fig. 2E). Blasting of the implants with AlO_2_ particles resulted in a relative homogenous, medium rough surface structure as observed at higher magnification (Fig. 2B–D, F–H). The surface profilometer measurements revealed a moderately rough surface with Sa value of 0.93 and 0.98 µm for the cylinder top and mesh, respectively (Fig. 2M). Depth profiling using AES showed an oxide thickness of 60 and 30 nm for the cylinder top and mesh, respectively (Fig. 2I, J, M). AES also revealed a predominance of titanium, oxygen and carbon on both the cylinder and the mesh surfaces with high amount of aluminium and minor contamination by natrium, potassium, kalium and copper (Fig. 2M). The subsequent energy dispersive X-ray (EDX) analysis showed that aluminium and oxygen coincided in certain areas on the surface (Fig. 2K, L). The appearance of the Al–O rich areas suggests them to be blasting residues embedded in the surface.

### Stoma port construction

Stoma port implantation and ileal reservoir construction were successful in all eight dogs. Seven dogs were fit for conversion to a functioning ileostomy (*n* = 4) and urostomy (*n* = 3), respectively. The seven ports were implanted for three-28 weeks (median, 13 weeks). One port had to be removed before conversion due to the formation of an abscess. The postoperative course and findings at autopsy are summarised in Table 1.

**Table 1:** Postoperative course and findings at the time of autopsy

ID no.	Model	Follow-up (weeks)	Connected (weeks)	Complications	Reason for explantation
EBM07	Ileostomy	4	1	Excessive ileal output causing fluid loss. Skin granulation.	Subileus (stenosis)
EBM09	N/A	4	0	Skin granulation	Abscess, implant lost
EBM10	Ileostomy	13	8	Excessive ileal output causing fluid loss. Skin granulation. Retraction of efferent ileum.	Subileus (Trichobezoars)
EBM11	Ileostomy	3	0.5	Skin granulation. Retraction of efferent ileum.	Fistula
EBM12	Ileostomy	9	4	Excessive ileal output causing fluid loss. Skin granulation.	Subileus (Trichobezoars)
EBM13	Urostomy	14	9	Sinus The skin around the device was wounded by the pressure of the healing device. Recurrent skin infections. Acute cystopyelitis.	Acute cystopyelitis
EBM14	Urostomy	20	16	Sinus Ileal ischaemia.	Ischaemic stoma
EBM18	Urostomy	28	18	Trauma to stoma. Skin granulation. Retraction of efferent ileum.	Planned

### Continent ileostomy group

#### Clinical results

The four animals in the ileostomy group were followed for a median of seven weeks (range, three-13 weeks) and conversion to a functioning continent ileostomy was performed after a median of 4 weeks (range, three to five weeks). The experiments were terminated in all four dogs due to complications, i.e., intestinal obstruction or anastomotic stricture or fistula formation. All implants remained stable in the abdominal wall, but recurrent superficial infection appeared around the port, causing granulation. The efferent segment retracted 3-10 mm inwards into the port during the observation time. An example of the macroscopic appearance is shown in Fig. 1C.

#### Histological results

**Fig. 3:**
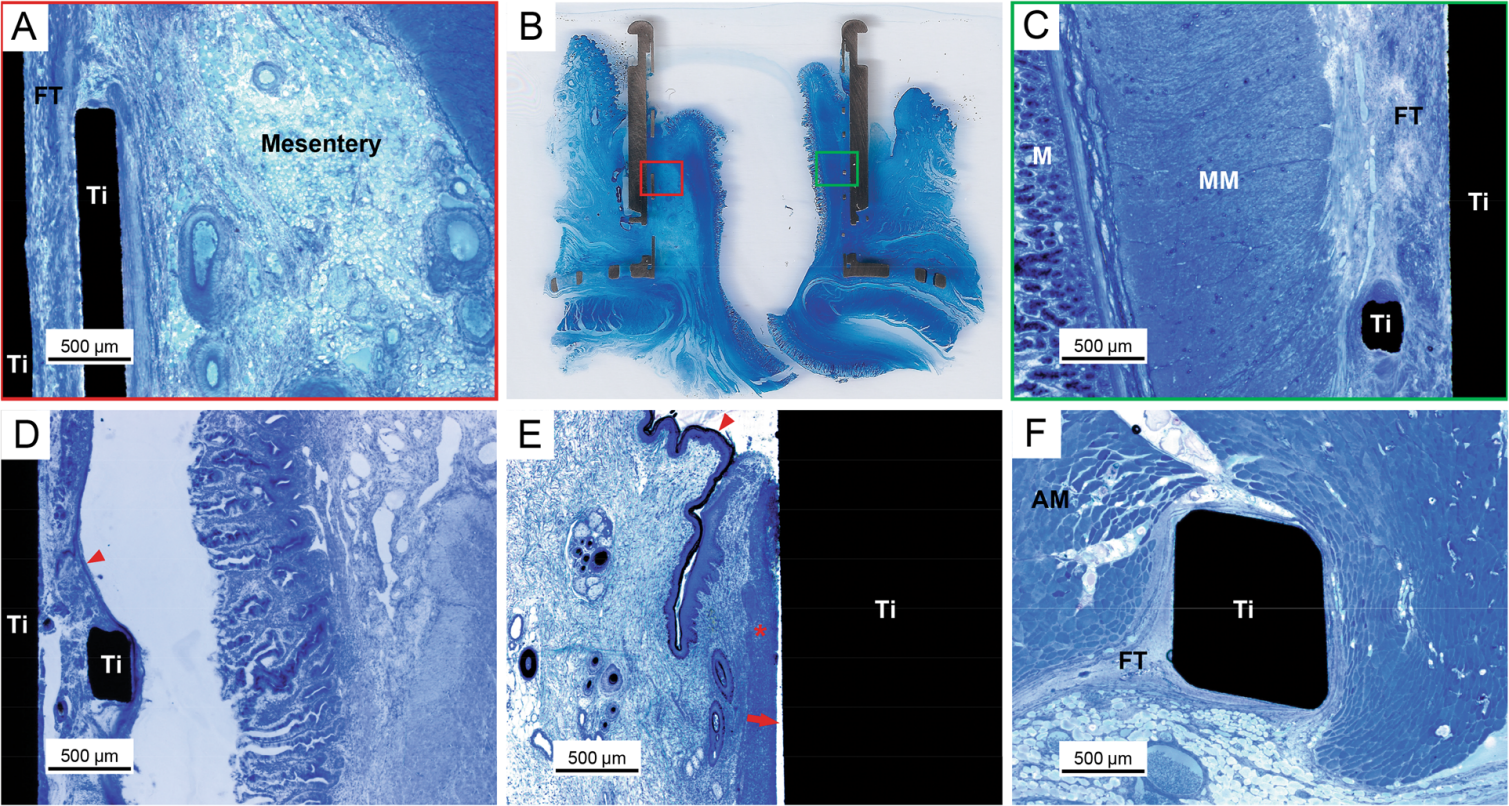
**A**–**C** Light micrographs showing the integration between the intestine and the inner mesh and cylindrical surface of the titanium (Ti) port in an ileostomy model (EBM12, 9 weeks). **A** On the anti-mesenteric side, integration was observed between the external part of the intestine and the inside of the titanium cylinder and the mesh. The vascularised fibrous tissue (FT), without signs of inflammation, filled the area around the mesh structure and merged with the outer tissue of the ileum. No major mucosal downgrowth was detected. M = intestinal mucosa, MM = muscularis. **C** On the opposite side, the mesentery ran along the mesh and integrated with the vascularises fibrous tissue (FT) around the mesh. No inflammation was detected. **D** Light micrograph showing the absence of integration between the intestine and the inner mesh and cylindrical surface of the titanium port (EBM10, 13 weeks). The intestinal mucosa (M) migrated downwards and separated from the material surface by material-adherent tissue containing numerous inflammatory cells and bacteria (red arrowhead). **E** Light micrograph of the skin-implant junction (EBM11, 3 weeks). The upper portion consisted of a well-keratinised epidermis (red arrowhead) that did not reach the outer surface of the port. No epidermal downgrowth was observed. In close vicinity to the implant surface, there was a zone of massive infiltration of inflammatory cells (red asterisk), mainly PMN cells and mononuclear cells, indicating subacute/chronic inflammation. A dense layer on the titanium cylinder indicated biofilm formation (red arrow). **F** Light micrograph showing the integration of the subcutaneous anchor placed in the abdominal muscle tissue (AM) (EBM14, 20 weeks). The anchor was well incorporated with connective tissue growing through the perforations. The vascularised fibrous tissue (FT) consisted of collagenous bundles with different orientations, forming a capsule around the titanium (Ti). No accumulation of inflammatory infiltrates was observed

The histological results in the continent ileostomy group are summarized in Table 2. One dog (EBM12) displayed a tight adaptation between the cylinder, mesh and the external portion of the intestine in the stoma port (Fig. 3A–C). Histology demonstrated a low degree of inflammation and no bacterial colonisation on the implant surface. In this case, the (vertical) retraction of the ileum was limited (3–5 mm). The integration between the mesh and intestine was incomplete in the other three dogs. In one dog (EBM10) (Fig. 3D), downward migration of the intestinal mucosa and implant-adherent inflammatory tissue and bacteria was demonstrated on both the mesenteric and anti-mesenteric sides. This resulted in the absence of integration between the intestine and the mesh inside the titanium cylinder at the time of study termination. Similar findings were observed on the anti-mesenteric side in the remaining two cases (EBM07 and EBM11). However, in these latter two cases, integration was observed on the mesenteric side, with mature connective tissue with few inflammatory cells filling the voids in the mesh and closely adapting to the titanium surfaces. In these specimens, the ileum retracted less on the mesenteric side than on the anti-mesenteric side.

**Table 2:** Histological results in the ileostomy group

ID no	Follow-up (weeks)	Connected (weeks)	Skin/implant junction	Ileum
EBM07	4	1	Granulation tissue. Subacute inflammation. Bacteria on the outer implant surface.	Mesenteric side: Integration, mature connective tissue with few inflammatory cells filling the voids in the mesh and closely adapting to the titanium surfaces. Anti-mesenteric side: No integration. Mucosal downgrowth. Infiltration of PMN cells and lymphocytes suggesting a bacterially driven inflammation.
EBM09	4	0	N/A	N/A
EBM10	13	8	Epidermis not in contact with implant. Granulation tissue. Subacute/chronic inflammation. Continuous biofilm on the outer implant surface.	Mesenteric and anti-mesenteric side: No integration. Mucosal downgrowth. Thick biofilm on implant surface. Mixed subacute-chronic inflammation (PMN cells present but mostly lymphocytes and macrophages).
EBM11	3	0.5	Tissue not in contact with implant. Moderate chronic inflammation. Bacterial growth and biofilm formation.	Mesenteric side: Integration, mature connective tissue with few inflammatory cells filling the voids in the mesh and closely adapting to the titanium surfaces. Anti-mesenteric side: No integration. Mucosal downgrowth. Infiltration of PMN cells and lymphocytes suggesting a bacterially driven inflammation.
EBM12	9	4	Moderate inflammation in the upper part. More inferior, dense, parallel connective fibres without signs of inflammation.	Complete integration on both the mesenteric and anti-mesenteric sides with moderate retraction.

*PMN* polymorphonuclear

**Fig. 4:**
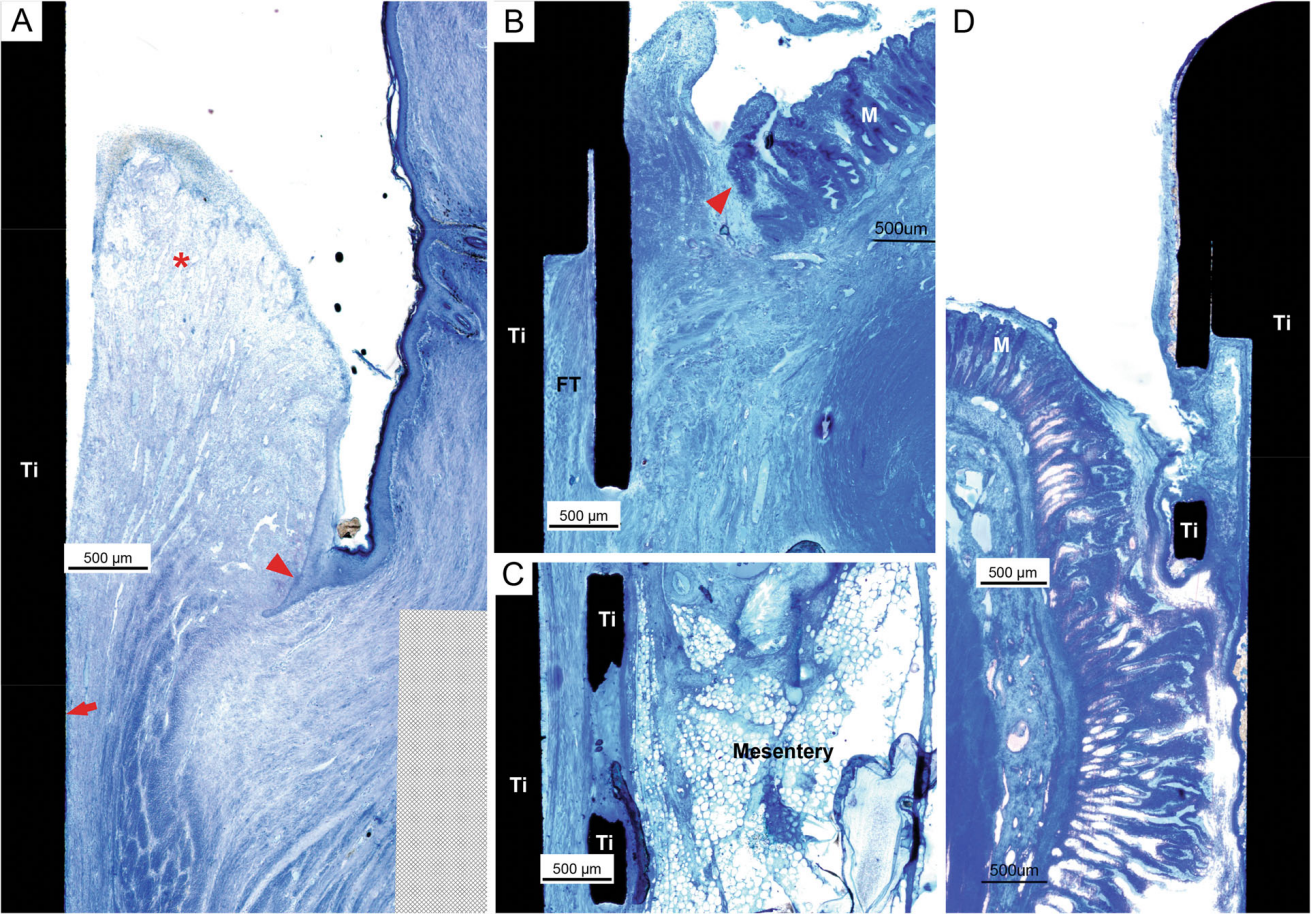
**A** Light micrographs of the skin-penetrating outer portion of the implant (EBM12, 9 weeks). The keratinised skin ends at a distance from the implant surface (red arrowhead). The space between the epidermis and the implant was filled by granulation tissue with moderate infiltration of inflammatory cells (red asterisk). At the junction between the soft tissue and the implant (red arrow), dense connective tissue parallel to the implant was well adapted to the material surface. **B**–**D** Light micrographs showing the integration between the intestine and the inner mesh and cylindrical surface of the titanium port in a urostomy model (EBM13, 14 weeks). **B** On the mesenteric side, integration was observed between the external part of the intestine and the inside of the titanium cylinder and the mesh. Vascularises fibrous tissue (FT) containing focal infiltrates of macrophages, PMN cells and lymphocytes was observed. No major mucosal downgrowth was detected (red arrowhead). M = intestinal mucosa. **C** Light micrograph further down in the cylinder showing integration between the mesentery and the vascularises fibrous tissue (FT) around the mesh. **D** Light micrograph illustrating mucosal downgrowth in the same sample on the anti-mesenteric side, as well as lack of integration between the intestine and the inner mesh and cylindrical surface of the titanium port (EBM13, 14 weeks)

In all specimens, the skin-implant junction was characterised by the absence of direct contact between the epidermis and the implant. Instead, granulation tissue was localised in the top portion between the epidermis and the outer surface of the implant. In a few specimens (Fig. 4A), close adaptation of connective tissue without inflammatory cells to the titanium surface was observed below the skin-implant junction. In most specimens, however, the main observation was the lack of integration associated with the presence of bacteria and a zone of inflammatory cells in close vicinity to the implant surface (Fig. 3E). Another prominent finding was the absence of skin epithelial downgrowth.

### Continent urostomy group

#### Clinical results

The animals in the urostomy group were followed for a median of 20 weeks (range, 14–28 weeks), and the left ureter was implanted in the reservoir after a median of 4 weeks (range, 4–10 weeks). An uneventful initial healing phase was followed by a stable period without clinical signs of pronounced inflammation or infection. A sinus tract (a small pocket between the skin tissue and the skin-penetrating part of the port), varying over time from almost zero to three mm, was observed with periods of granulation tissue growth. When the reservoir reached an adequate volume, the urostomy was made continent by gradually increasing the period of lid attachment to the implant (up to 5 h). Macroscopically, the external portion of the intestine in the stoma port seemed attached to the mesh inside the port in one of the cases. In two cases, the intestine was retracted below the implant top. The experiments were ended at different time intervals because of pyelitis or ischaemic efferent segments. An example of the macroscopic appearance during the experiments is shown in Fig. 1D.

#### Histological results

The histological results in the continent ileostomy group are summarized in Table 3. The subcutaneously located anchor part of the titanium port was well integrated and surrounded by fibrous tissue (Fig. 3F). On the mesenteric side, integration was observed between the external part of the intestine and the inside of the titanium cylinder and the mesh (Fig. 4B, C). The vascularised fibrous tissue contained focal infiltrates of macrophages, polymorphonuclear (PMN) cells and lymphocytes. No major mucosal downgrowth was detected. On the other hand, the anti-mesenteric side was characterised by complete mucosal downgrowth, exudates and no integration with the titanium port. The accumulation of bacteria as dense aggregates (biofilms) on the surface of the titanium was observed in two out of the three ports (Fig. 4D).

**Table 3:** Histological results in the urostomy group

ID no.	Follow-up (weeks)	Connected (weeks)	Skin/implant junction	Ileum
EBM13	14	9	Subacute inflammation with PMN cells, fibrous encapsulation. Epithelial downgrowth halfway down on the top cylinder.	Mesenteric side: Integration without inflammation. Anti-mesenteric side: Mucosal downgrowth, separation between ileum and port. Areas of exudate around the mesh.
EBM14	20	16	Epidermal downgrowth on anti-mesenteric side, cylinder covered with biofilm, inflammation in the tissue. Less inflammation more inferior. Mesenteric side. No epidermal downgrowth, no biofilm detected, but inflammation in the tissue closest to the metal (1–1.5) mm from surface. Tissue separated from the metal, possibly an artefact.	Mesenteric side: Integration. Loose fibrous tissue in mesh area. Inflammation close to ileum. Anti-mesenteric side: No integration. Biofilm formation.
EBM18	28	18	Limited epidermal downgrowth, areas with biofilm. Inflammation in the tissue closest to the metal (1–1.5) mm from surface.	Mesenteric side: Integration without inflammation. Slight mucosal downgrowth. Anti-mesenteric side: No integration. Mucosal downgrowth and separation from the mesh. Exudates. Areas with biofilm formation?

*PMN* polymorphonuclear

**Fig. 5:**
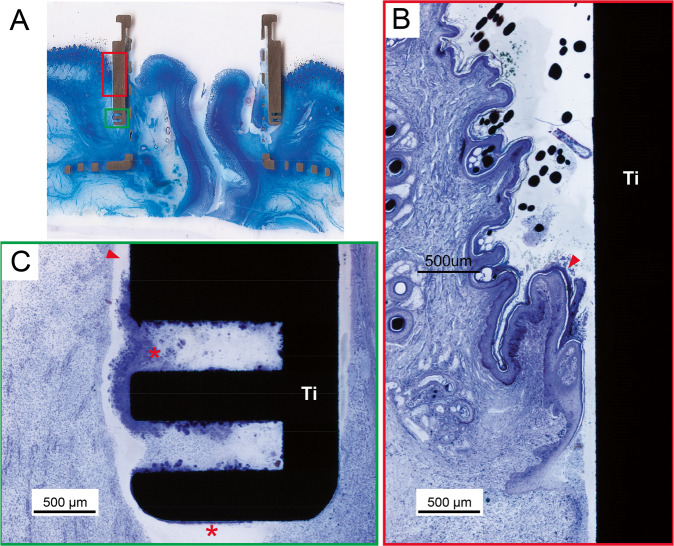
**A** Light micrograph demonstrating the lack of epidermis-titanium contact in the skin-penetrating part of the port (EBM18, 28 weeks). **B** Epidermal downgrowth (red arrowhead) along the titanium surface was observed. **C** Further down, there was no tight adaptation of fibrous tissue to the surface (red arrowhead), and focal areas with bacterial growth and biofilm formation were evident (red asterisks)

The outer surface of the titanium port facing the skin was not in direct contact with tissue and was characterised by varying degrees of epidermal downgrowth (Fig. 5A–C). Focal areas of biofilm formation were detected on the material surface.

#### Backscattered electron imaging

**Fig. 6:**
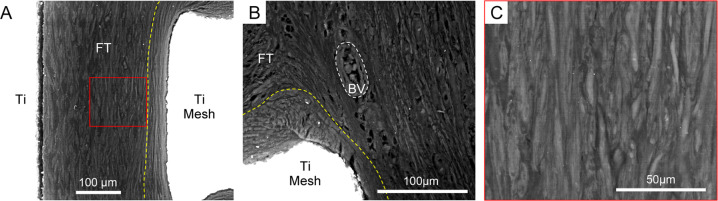
Backscattered electron imaging (BSE-SEM) of the interface between the external part of the intestine and the inner surfaces of the titanium implant in urostomy model (EBM13, 14 weeks; shown in Fig. 4C). Images were obtained following sample treatment with iodine in potassium iodide (Lugol) solution. **A** The inner mesh (Ti Mesh) and the cylinder (Ti) showing integration with the fibrous tissue (FT) at the anti-mesenteric aspect of the intestine. Yellow-dotted line depicts part of the dense fibrous capsule intimately in contact with the mesh (Ti Mesh). **B** Peri-implant fibrous tissue (FT) delineating the mesentery and showing vascular supply (white-dotted line; BV: blood vessel) in close vicinity to the dense capsule (yellow-dotted line) around the mesh (Ti Mesh). **C** Magnified area highlighting fibrous bundles with different orientations in tissues integrated with the inner cylinder shown in (**A**) (Red rectangle)

Imaging using back-scattered scanning electron microscopy (BSE-SEM) allowed to highlight the fibrous organisation in peri-implant tissues, as shown in Fig. 6. Fibrous bundles depicted a micro-scale adaptation to the implant surface and confirmed the histological evidence of intestine integration to the urostomy device (Fig. 6A, C). This tight adaptation was particularly visible at the periphery of implant mesh forming a dense fibrillar capsule. In addition, the visualisation of vascular components at the external aspect of the intestine further supported the blood supply furnished by the mesentery to the fibrous peri-implant tissue (Fig. 6B).

## Discussion

A prerequisite for a functional skin-penetrating implant with an enterostomy is a structural barrier between the intestinal constituents and the surface of the implant. If bacteria and faecal debris are allowed to penetrate between the intestine and the implant, this barrier is disrupted. Although the present results do not provide a solution to achieve predictable integration, our findings indicate that it is possible to achieve integration between the inner surface of a titanium port with a cylindrical mesh design and the intestine. At present, we have no information about the spatial or temporal repair processes leading to this result. Nevertheless, it will be of future interest to explore the cellular and microbiological processes behind this soft tissue repair. By employing cellular and microbiological techniques, such as qPCR, culturing and bacterial profiling, on soft tissue samples retrieved using paper points and micro-biopsies retrieved at and around the BAHS, insight into these processes has recently been gained. Despite being normally healed and functional, the BAHS was shown to be associated with prolonged inflammatory and remodelling activity, together with bacterial growth, despite the absence of macroscopic signs of inflammation or infection [27–29]. However, it is important to recognise that the site (retro-auricular in the temporal bone versus abdominal), anchoring type (bone anchored versus soft tissue anchored) and implant size differ between BAHS implants and the stoma port evaluated in the present study.

Other techniques for creating continent enterostomies using either external removable devices or implants have been reported [1, 4, 30]. However, in none of these studies was the bowel wall allowed to grow into the device. Bustamante et al. used vitreous carbon polymer implants to create a connection between the abdominal wall, bowel wall and implant in dogs [5]. In urostomy applications, devices made of titanium or carbon-based materials have been implanted to create continence [3, 10, 31]. However, most of the implanted devices were removed due to infection, fistula formation or poor ingrowth and function. Jonsson et al. used titanium rings to stabilise dysfunctional nipple valves in continent urostomies [10]. The rings attached to the serosal surface of the ileum with only minor signs of inflammation. In the latter application, the aim was to use the titanium ring to create an attachment between the reservoir wall and peritoneum to stabilise the nipple.

From applied and clinical points of view, additional observations in the present study might be of importance. Histology and electron-microscopy findings showed that integration between the titanium port and intestine was more commonly observed in the mesenteric region of the intestinal circumference. The latter observation is supported by the development of a fibrotic capsule tightly adapted to the implant surface, both the cylindrical part and the mesh. Fibrotic encapsulation is a peculiar feature of the end-stage response of soft tissues to Ti-based implants, and sequentially take place following phases of inflammation and tissue repair [32]. The intimate disposition of the capsule around titanium suggests that fibres are deposited in an incremental fashion at the interface, and undergo guidance by the device contour. In contrast, mucosal downgrowth and bacteria growth in the form of implant surface biofilms were both prominent findings in situations with a non-integrated state. Interestingly, this was more commonly associated with the anti-mesenteric portion. At present, it is difficult to determine the causes of these observations. Although speculative, possible explanations could be differences in vascularisation and mechanical stresses. Initially, the blood supply is higher on the mesenteric side, but histological observations reveal that with time, there is also neovascularization in the subcutaneous tissue on the anti-mesenteric side. Mechanical stresses exerted on the tissue-material interface and intestinal peristalsis might also play an adverse role in the ingrowth process [33, 34]. Furthermore, the ileal reservoirs were intubated several times per day, causing traumatisation, during the experiments. The mesenteric fatty tissue might serve as a cushion, thereby protecting against repeated mechanical stresses at the tissue-mesh interface caused by catheterisation, a factor that might favour ingrowth on this side of the port.

A second prerequisite for the safe and functional combination of skin-penetrating implants and an enterostomy is the formation of a structural and functional barrier between the skin and implant. An important finding in the present study was the difficulty of achieving skin-implant integration, i.e., epidermis-titanium contact and tight adaptation of fibrous tissue, in both the ileostomy and urostomy groups. In the ileostomy group, the morphological evaluation demonstrated granulation tissue and the presence of biofilms, whereas in the urostomy group, varying degrees of epidermal downgrowth and biofilm formation were prominent findings. An important cause of implant failure is marsupialisation, which represents the final result of epithelial downgrowth along an implant surface penetrating the skin barrier [35]. Various implant designs and implant surface properties have been tested to prevent the downgrowth of epithelial cells, mostly with limited success [36–39]. Epithelial downgrowth allows bacterial invasion to occur between the skin and the implant interface, which often results in infection [40–42].

Given the importance of competition for the surface of implanted foreign materials between different cell types and microorganisms during the initial critical phase of soft tissue repair, the provision of an optimal healing environment at the implant surfaces needs careful attention [43]. Since only one of the dogs revealed complete integration at both the inner (titanium-intestine) and outer (titanium-skin) interfaces, it is likely that the formation of a structural and functional inner barrier is required to allow healing of the outer barrier. Measures to minimise bacterial contamination at these interfaces during healing are therefore important. Physical and chemical surface properties play an important role in the development of implant-associated infection. In the current study, part of the material/bio interfaces were colonised by microorganisms and a complete or partial lack of integration was evident. While the biological and chemical interfaces between titanium and bone have been well characterised, significantly less is however known about the interface formed between titanium and soft tissues of various types. A close contact between implant and soft tissues in combination with a thin surrounding soft tissue capsule with low number of inflammatory cells and oblique collagen orientation is regarded as optimal [44, 45]. However, implant surface structures that promote cellular attachment are also easily colonised by invading pathogens. To prevent this bacterial colonisation, surfaces with antimicrobial functionality or antimicrobial-releasing surfaces have been proposed. Hence, a multifunctional design of the implant surface, promoting tissue integration while at the same time preventing bacterial contamination and colonisation, is desired, particularly in demanding applications such as the implant system studied here [46]. The different parts of the implants (cylinder, mesh and anchor) used in this study had similar chemical and morphological surface properties exhibiting an increased oxide thickness, compared with native titanium, and a smooth to moderately rough surface. Even though the implant design could partially explain the beneficial response at and around parts of the implant, the study does not provide any mechanistic insights to the cause and effect behind the observed tissue responses and its relation to the chemical and physical characteristics of the implant surface.

Moreover, efforts to reduce tissue mobility in relation to the percutaneous implant are of importance since mechanical stress at the skin-implant interface, generated by relative movement between the implant and skin, is another cause of percutaneous implant failure [33]. When percutaneous implants are anchored in bone (e.g., the BAHS), close adaptation of the epidermis and dermis to the abutment is achieved [47]. Although this should be regarded as an incomplete structural barrier, the surrounding host defence provides an additional functional barrier in situations with infection and breach of the structural barrier [42, 48]. A local, and possible permanent, change in the stomal microbiota following the implantation of percutaneous, bone-anchored prostheses in humans has been demonstrated, both for limb prostheses [19, 49] and the BAHS [27, 29]. These studies confirmed colonisation by pathogens, typically *Staphylococcus* spp., at the implant sites, and often, these findings were independent from traditional clinical signs of infection [19, 27]. However, such an infected skin-implant interface is amenable to successful treatment and rarely results in device removal, although recent observations indicate that biofilm formation on the implants themselves may be more prevalent than previously communicated [50, 51].

Due to the animal model used in the present study, with the dogs running and jumping soon after surgery, the stress at the skin interface was substantial. Further studies are therefore needed to counteract the interfacial stress associated with non-bone-anchored, percutaneous devices, e.g., by improving the port design. To facilitate the development of structural and functional barriers between the implant and soft tissues, prevent bacterial contamination and reduce the mobility between the implant and tissues, future strategies include improvement of the surgical procedures, titanium port design, stoma maintenance and animal models.

## Conclusion

The results of the present study indicate that a titanium implant may be used as a port for the creation of a continent ileo- or urostomy. Nevertheless, to create a predictable, long-term solution for human treatment using this principle, it is necessary to improve the current techniques. Major strategies include reducing tissue mobility by optimising the surgical procedures, port design and accessories, exploring 3-stage procedures to allow complete integration, and reducing/delaying the postoperative bacterial burden.

## Data Availability

Data is available on request.
